# Characteristics of successfully implemented telemedical applications

**DOI:** 10.1186/1748-5908-2-25

**Published:** 2007-07-27

**Authors:** Aud Obstfelder, Kjersti H Engeseth, Rolf Wynn

**Affiliations:** 1 Norwegian Centre of Telemedicine, University Hospital of Northern Norway, Tromsø, Norway; 2 Department of Nursing and Health Science, University of Tromsø, Tromsø, Norway; 3 Department of Clinical Psychiatry, University of Tromsø, Tromsø, Norway

## Abstract

**Background:**

There has been an increased interest in the use of telemedical applications in clinical practice in recent years. Considerable effort has been invested in trials and experimental services. Yet, surprisingly few applications have continued beyond the research and development phase. The aim of this study is to explore characteristics of successfully implemented telemedical applications.

**Methods:**

An extensive search of telemedicine literature was conducted in order to identify relevant articles. Following a defined selection process, a small number of articles were identified that described characteristics of successfully implemented telemedical applications. These articles were analysed qualitatively, drawing on central procedures from Grounded Theory (GT), including condensation and categorisation. The analysis resulted in a description of features found to be of importance for a successful implementation of telemedicine. Subsequently, these features were discussed in light of Science and Technology studies (STS) and the concept of 'social negotiation'.

**Results:**

Telemedical applications introduced into routine practice are typically characterised by the following six features: 1) local service delivery problems have been clearly stated, 2) telemedicine has been seen as a benefit, 3) telemedicine has been seen as a solution to political and medical issues, 4) there was collaboration between promoters and users, 5) issues regarding organizational and technological arrangements have been addressed, and 6) the future operation of the service has been considered.

**Conclusion:**

Our findings support research arguing that technologies are not fixed entities moving from invention through diffusion and into routine use. Rather, it is the interplay between technical and social factors that produces a particular outcome. The success of a technology depends on how this interplay is managed during the process of implementation.

## Background

One of the more significant developments over the past decades has been the emergence and widespread deployment of information and communication technologies (ICT) [1-3]. The 'digital revolution' has transformed our everyday lives and has had a pervasive influence on work and organizations. ICT has captured the attention of health care providers as well as health policy makers, who are encouraging these technologies primarily because of their potential to address issues such as inequalities in access to health care and the need to reduce costs, while delivering at least equivalent, if not better, standards of health care than traditional alternatives [4-8].

Telemedicine is the use of information technology to support delivery of health care from a distance [6]. Despite the general impact of new technologies in society and the political will to promote telemedicine in public health care, telemedicine has primarily been used on a small scale in clinical activity. As a field of practice, telemedicine is mainly characterized by trials, demonstrations, or experimental services that do not endure beyond the life of specific research and development projects. Only a few telemedical applications have been implemented on a wide scale and sustained [9-14].

It is commonly suggested that the main reason for the low routine clinical use of telemedical applications is the insufficient evidence of its efficacy, in terms of both clinical and organizational impact on the health care sector. Without evidence of any effect, professional and political support for telemedicine cannot be sustained [12,13,15]. However, the outcome of clinical trials does not communicate the whole story about what is needed to make telemedical applications work [15]. Conditions operating during the projects, which could have been important for the outcome of the trial, are seldom mentioned or questioned; neither is the correlation between positive outcomes of clinical trials and routine use. In addition, it is often unclear in the literature whether the telemedical application being discussed has been introduced into routine operation, and whether any such routine operation in fact is an extension of the research and development project or represents a new introduction of the application in a completely different part of the health service.

An understanding of why telemedicine is seldom used in clinical practice is important to clinical and policy proponents of these technologies, who see telemedical applications offering solutions to some key problems in improving access to health care and equitably distributing specialist clinical expertise [12]. Previous telemedicine research provides little insight into why there is so little routine use of telemedicine in clinical practice. In recent years, however, studies on 'organizational issues' have emerged [7,12,13,15,16]. Some of the reports refer to the study of science and technology in a social context, or STS theory [17,18], and the term 'social negotiation' [17] has been used to describe a key aspect of the difficulties of routine clinical use. The basic assumption in STS theory is that scientific knowledge and technologies do not evolve in a vacuum. Rather, they should be seen as parts of the social world, being shaped by it, and simultaneously shaping it. The term 'social negotiation' indicates that while technological issues such as inadequate design or poor performance will reduce the system's chances of being implemented successfully [19], use or non-use is determined by the social context in which the technology is implemented. This is because a more or less explicit controversy will always arise when a new technology is implemented. The controversy may involve the problems that the technology is intended to solve, as well as the ways in which they are to be solved. The various participants may also have different perceptions of what the organizational challenges are, what the solution should look like, and whether an implementation is viewed as a success or a failure.

From the perspective of those who question the assumption that insufficient evidence of telemedicine is the reason for low clinical use, it is clear that new technologies alone do not create change. Rather, it is the interplay between technical and social factors that produces particular outcomes [17-19]. That is, organizational difficulties lie in the deep interrelation of technical and social aspects of designing and implementing technologies. Conversely, success entails handling these complex, heterogeneous factors, which are expressed in controversies and solved through social negotiation.

The purpose of our study is to contribute to this emerging literature on telemedicine and organisational issues by doing an in-depth analysis of the characteristics of telemedical applications that have been implemented into routine clinical practice. Our method is a review of the literature on telemedicine with success. Our principal research questions are: 1) What are the characteristics of telemedical applications that have successfully been implemented in routine clinical practice? 2) In what way are these characteristics associated with the emerging literature on telemedicine and organisational issues, particularly with respect to STS theory and the concept of 'social negotiation'.

On the basis of our findings, we aim to suggest how proponents of telemedicine should proceed when planning to implement telemedicine in clinical practice, and to indicate areas for further research.

## Methods

### Overall research design and method

An extensive search of telemedicine literature was conducted in order to identify relevant articles. In selecting the articles to be included in the study, we initially searched a range of databases using specific key words. The search was subsequently refined, and the number of articles reduced, by excluding less relevant literature. The procedure followed is described in more detail below. Thus, a small number of articles were identified that described characteristics of successfully implemented telemedical applications. We found that our research questions, as well as the type (*i.e.*, mainly qualitative) and small number of articles describing successfully implemented telemedical applications, would be best approached with a qualitative method. We analysed the data with a qualitative method drawing on central concepts from Grounded Theory (GT), including condensation, categorisation, and data saturation [20-22]. The analysis resulted in a description of features found to be of importance for a successful implementation of telemedicine. Subsequently, these features were discussed in more detail in light of STS theory and the concept of 'social negotiation' [17-19].

### Strategy for the database search

The research on telemedicine is interdisciplinary, and dominated by demonstrations, feasibility, and evaluation studies [23]. We expected few articles describing the successful implementation of telemedicine, as well as a wide variety of keywords. Thus, we performed a broad initial research. The following electronic databases were searched: Cochrane, PubMed, Web of Science (ISI), TIE, sociological abstracts (Cambridge), ERIC, PsychInfo, and CINAHL. The keywords used were: assessment, evaluation, utilization, case, clinical application, difficulties, barriers, challenge, critical issues, facilitators, limitation, prevention, success, failure, diffusion, dissemination, adoption, meta-analysis, review and telehealthcare, telemedicine, e-health. To validate the keywords used in the initial search, we developed a list of possible keywords by reading through articles of telemedicine studies from personal archives. We performed a test search of all the keywords and those with hits on more than 1,000 were deleted. References of the included studies and citations were not searched.

### The process of exclusion and inclusion of studies

**Figure 1:**
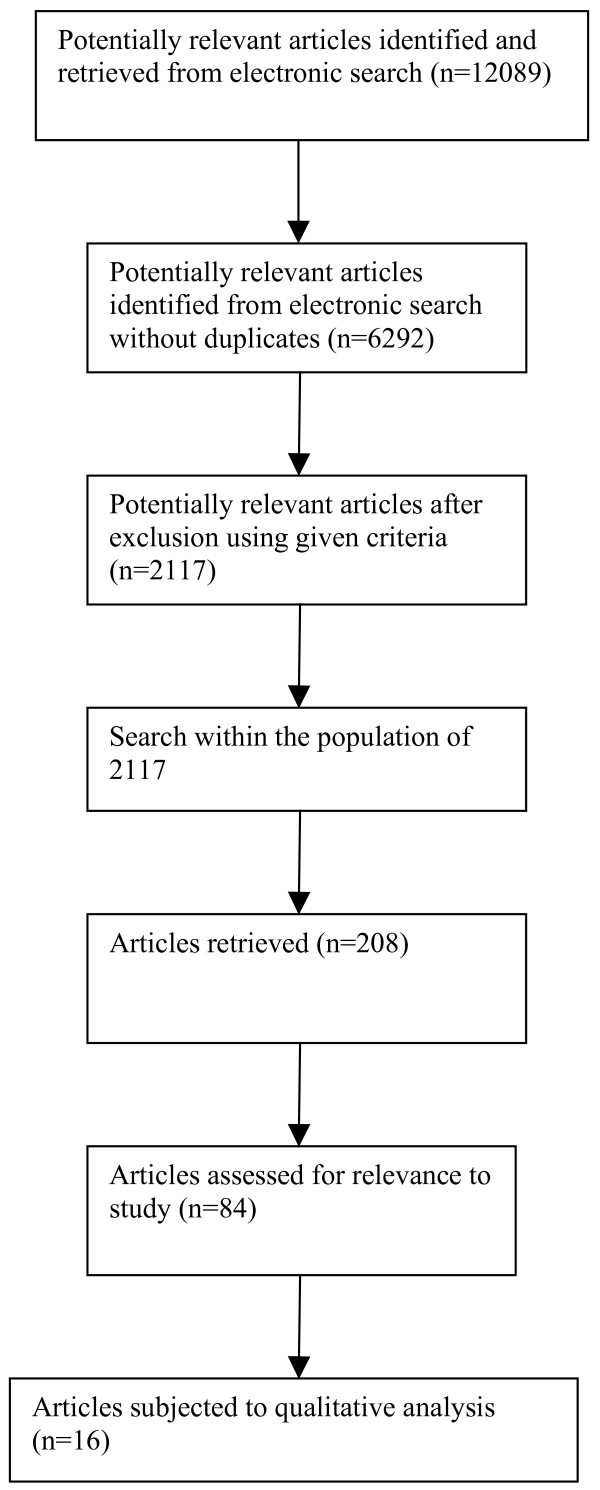
Selection process for studies included in analysis.

Despite performing a test search, the retrieved number of articles after the initial search was vast (Figure 1: n = 12089). We reduced the sample of article further by excluding articles written in a language other than English and those published before 1990. Studies in which the use of telecommunication technologies was primarily for educational and administrative purposes and not linked directly to patient care, as well as studies in which the patient was not physically present at either point of care, were also excluded [23]. Studies on telephone consultation services, internet services where no communication between professionals and/or professionals and patients was possible, and review or discussion papers were also excluded. In addition, if any single study resulted in multiple publications, we included only the principal article, focusing on the clinical use of telemedicine. Finally, all studies that had no abstracts when retrieved from the original electronic databases were excluded (Figure 1: n = 2117).

To assess the remaining studies for relevance, we first browsed the title and abstract on each article. However, we quickly realised that a separation of studies of successfully implemented telemedicine from other descriptive studies – in particular, evaluation and outcome studies was not possible. A more comprehensive reading of all the articles was required, which was something we could not do within the frame of the present research project. Thus, we further reduced the selected articles by conducting another search within the sample looking specifically for empirical studies on telemedicine in clinical use (Figure 1: n = 208). The keywords used at this stage were evaluation, outcome, and implementation. To determine the remaining studies potential suitability for the present study, the title and abstract of each article, were read by all three authors. The full articles were then assessed for relevance (Figure 1: n = 84). The articles were subsequently sorted into the following main categories: 'clinical use', 'outcome study', 'uncertain', and 'background'. The inclusion criteria for studies in the 'clinical use' category were that the authors of the studies categorized the applications as such. In addition, the articles had to document some type of activity in the described telemedical service.

### Qualitative data analysis

The articles in the 'clinical use' category were analysed further qualitatively, drawing on central concepts from GT [20,21,24,25], The next step in the analysis was to identify (condense) common concepts and features in the articles relevant to the present study, and then cluster these concepts and features in linked themes (categorization). That is, on the basis of similarities and dissimilarities in the content of the articles (data), relevant categories that included concepts and features of similar meaning were elicited from the articles. Next, in order to describe the properties of each category, the concepts and experiences assigned to each category were explored further. The content of the articles was categorized throughout the research process. As new data emerged, the categories were tested and refined until 'data saturation' [20,22] occurred, *i.e.*, until the categorization process did not give us new insight about characteristics of successfully implemented telemedical applications. As the research progressed, memos were produced summarizing findings and explanations. These memos were subsequently used to produce the results presented below.

## Results

### Overview

**Figure 2:**
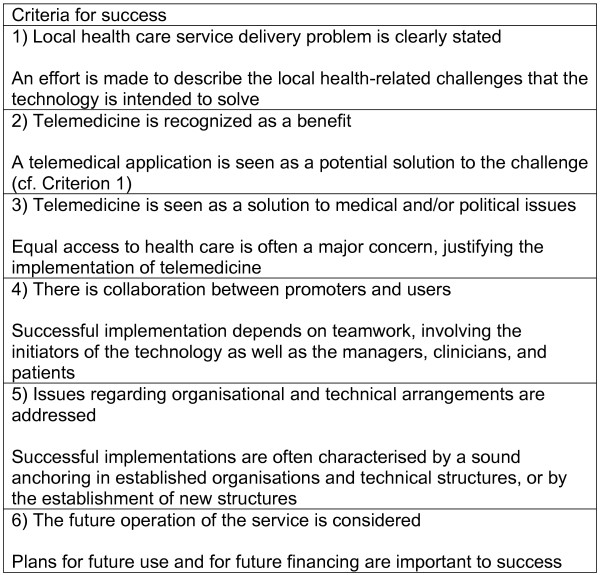
Criteria for success of telemedical applications.

Of the more than 12,000 articles initially retrieved, we identified only 16 studies of telemedical applications in clinical use. Following the analysis, we found certain general characteristics of these applications, which we have described in terms of six main categories (Figure 2). These categories are: local health care service delivery problems are clearly stated; telemedicine is seen as a benefit; telemedicine is seen as a solution to political and medical issues; there is collaboration between promoters and users; issues regarding organisational and technical arrangements are addressed; and the future operation of the service is considered.

Examples of how these common features are reflected in successful telemedical trials appear below. Categories one, two, five, and six are presented together in the same section. Please refer to the Appendix (Additional file 1) for a more thorough presentation of each of the articles discussed.

#### Local service delivery problems are clearly stated and telemedicine is seen as a benefit (categories one and two)

In all the studies of telemedicine in routine use, the local medical and health-related challenges that the technology is to solve are clearly described. The medical challenges that are described vary from acute and chronic diseases to disabilities. For example, La Monte *et al. *[26] describe how telemedicine is used to provide optimal acute stroke treatment where local specialists are not available. The study shows how an emergency department physician is linked with a specialist in a stroke department centre, and how this consultation provides an opportunity for administration of thrombolytic drugs within the short therapeutic time window (approximately three hours) associated with ischemic stroke. An example of a study in which telemedicine is used to handle challenges associated with chronic and medically complex conditions is described by Gray *et al. *[27]. The authors show how Baby CareLink, a multifaceted telemedicine application, can provide enhanced educational, emotional, and medical support to families of high-risk newborns both during their hospitalization and following discharge. Another example is provided by Ono and Lindsey [28], who describe how a children's hospital is using telemedicine to provide and coordinate care to children with paediatric orthopaedic conditions and chronic burn scars in 22 hospitals on the Pacific islands. A final example is Moses *et al. *[29], who point out that conditions for performing routine endoscopies in rural areas are poor and illustrate how a tele-endoscopy service supports primary care physicians performing endoscopies.

#### Telemedicine is seen as a solution to political and medical issues (category three)

Reasons provided in support of telemedicine implementation include its role in efforts to solve local medical and health-related challenges, as well as the health policy objective that citizens should have the same access to health services regardless of their geographical location. A typical example is Gulube and Wynchank [30], who described the development and implementation of a large telemedicine programme in South Africa. The programme is based on the government's wish to compensate for the negative consequences of previous political rule, which *inter alia *led to an unacceptable concentration and inappropriate distribution of health practitioners and expertise. Today, health care and expertise are concentrated in the major urban centres, while people living in rural areas have limited access to basic health care because of geographical isolation and poor public transport.

In many studies, the argumentation related to health policy is supported by descriptions of the unfortunate consequences that may arise if particular groups who live in outlying districts do not receive health assistance or other support. The descriptions of the consequences are based on relevant medical research and the rights of various interest groups. Examples of studies that refer to medical research include Moses *et al. *[29], Chan *et al. *[31], LaMonte *et al. *[26], and Lawton *et al. *[32].

There are only two studies [29,31] that legitimize the implementation of telemedicine by referring to evaluations of previous pilots, including analysis of costs, user satisfaction, and clinical outcome. Moses *et al. *[29], in particular, maintain that it is not sufficient to justify implementation of telemedicine by referring to local challenges, health policy, and medical research. All implementation trials must be based on evaluation results that demonstrate diagnostic quality of the images, costs, and provider/patient satisfaction. This enables organisations to make informed choices prior to the investment of significant time and resources. In contrast, the other studies state that evaluation results will be reported in future publications. Evaluation activities have been performed during these trials, but sample sizes were too small to assess any outcome variables.

Two further arguments are used to justify the local implementation of telemedical applications. The first argument is that a general transfer of competence takes place from the specialist health service to the local health service. For example, in the case of telemedicine for stroke [26], this general transfer of competence has led to improved abilities of the community hospital to recognize stroke and increase the speed of basic care. Another study emphasizing telemedicine's role in transfer of competence is DeLieto *et al. *[33]. The second argument is a reservation about the benefits of the technology, and states that ICT should not replace face-to-face medical practice, but rather provide an additional tool to complement current health care services [28,35].

#### There is collaboration between promoters and users (category four)

Promoting acceptability of the new telemedical applications and adapting the technology to the requirements of the health care service are key aspects in all of the studies of successful telemedicine. Acceptability and adaptation are promoted through a close dialogue between the initiators of the technology and its users throughout the trial phase. Users of the technology include individuals at management level, clinicians, and patients. The dialogue is both informal and formal. The informal dialogue emerges through the presence of the researchers, project managers, and system developers in the local context in which the technology is to be implemented. This presence enables the various participants to become acquainted with each other's work and knowledge, and thus establishes a basis for developing a mutual understanding of the challenges, as well as the solution to the problem. The formal dialogue takes place *inter alia *through the creation of local project groups for planning and implementation, through users' participation in developing the goals and methods of practice, and their involvement in planning and carrying out the implementation. Organization of training in the use of the technology, implementation of in-depth evaluation studies and communication with government agencies represent other more formal means of interaction.

Doolittle [36], Chau and Hu [37], and Khoja *et al.*[38] provide good examples of promoting acceptability of telemedical applications. In different ways they point out that the ultimate success of telemedicine as a viable alternative for service delivery and collaboration requires that organizations implementing the technology address technological and managerial challenges. Another good example of a study focusing on adoption and acceptability relates to Baby CareLink [27]. It is not explicitly stated that this type of activity is a criterion for success, but the study provides a detailed description of the way in which general internet technology is adapted to the needs that the service has been promised to fulfil. The needs are built into the system architecture, which implies a sound knowledge of the field of practice and thus a high level of informal involvement. A final example is LaMonte *et al. *[26], which effectively illustrates how user involvement and participation is accomplished. The process for involvement and adaptation in that study is described more explicitly than in Gray *et al. *[27], but there is no reference to involvement as a criterion of success.

#### Issues regarding organisational and technical arrangements are addressed and the future operation of the service is considered (categories five and six)

Descriptions of how the new telemedical applications has become grounded in organizational and technical arrangements, whether this is in established structures or in new ones created as a result of telemedicine, are striking in studies of successful telemedicine. For example, in the study of telemedicine for stroke [26], we see that the telemedical application emanates from a telephone consultation service between a team of specialists in stroke treatment and community hospital emergency departments throughout the state of Maryland. The study is a description of the way the emergency department of one community hospital replaced telephone consultations with a telemedical application. Other examples of how the telemedical applications become anchored in existing structures are Baby CareLink [27] and tele-endoscopy [29]. Baby CareLink was implemented as a part of a comprehensive telemedicine network for the neonatal intensive care unit and tele-endoscopy as a component of a state-wide health information network.

Other studies reflect a stronger emphasis on establishing formal routines for the use of telemedical applications. LaMonte *et al. *[26] describe the establishment of fixed routines for training in the use of the technology in response to frequent changes in staff and episodic refresher courses for permanent staff. Gulube and Wynchank [30] point out the importance of establishing guidelines for how to use the system. Gray *et al. *[27] describe the importance of establishing arrangements for maintainability of the system. In one study, a new position was even created to facilitate use of the telemedical application [39].

In all the articles, we see assertions that implementing the telemedical application in routine use requires service financing and that, at a minimum, a programme must be economically accountable for operating costs incurred in service delivery. Only a few authors, however, such as Chau and Hu [37], refer to establishment of routines for financing use of the application.

## Discussion

We will now discuss our findings from the qualitative analysis, *i.e. *the typical characteristics of successfully implemented telemedical applications, in relation to the concept of 'social negotiation'. We begin with a general discussion of what type of negotiations the project managers have to deal with during the process of implementation. Then, a more detailed discussion of each of the categories presented in the results section, with an emphasis on categories number four, five, and six, will follow. Finally, we will discuss some aspects of our method, and present our recommendations for how to proceed when planning to implement telemedicine in clinical practice.

### The concept of 'social negotiation' and the successfully implemented telemedical applications

When new technology is implemented, a controversy about which problems the technology is to solve, as well as the ways in which they are to be solved, will typically arise [17-19]. The various participants is involved in implementation of the technology may have different perceptions of what the organizational challenges are, what the solution should look like, and whether an implementation is to be understood as a success or a failure. Technological success entails handling these controversies through social negotiations. Our research revealed that in clinical settings where telemedical applications are in routine use, such controversies have been handled during the implementation phase. Because telemedicine is a field of applications that are not clearly defined at the outset [40], but need to be specially designed for use in different and particular medical specialities, this point is of special importance. In the studies of successful telemedical applications, the disagreements, concerns, and discussions are not often described explicitly. Rather, there are descriptions of the problems that the technology is to solve, and the way in which the various participants have worked together throughout the implementation project precisely to reach an agreement on the way in which the technology can contribute to solving the problems. Because these applications have been successful, we must assume that the participants in question have managed to reach agreement. That is, what we have seen in our study is that the applications and the fields of practice have been treated as dynamic units that have undergone a process of mutual adjustment, via the process of implementation. Alternatively, we can say that the project management to a great extent has accepted the inevitable uncertainty accompanying every technology implementation project, balanced carefully between initiating organizational change and drawing upon technologies as a change agent without attempting to pre-specify and control this process [19].

### The characteristics of successfully implemented telemedical applications

#### Local service delivery problems are clearly stated and telemedicine is seen as a benefit (categories one and two)

Most of the studies of the successful telemedical applications have described how the applications are used to solve specific local problems of a medical, technical, or organisational type. As the types of problems in which telemedicine is seen as a solution vary greatly, we believe that telemedical applications may be of use in many, if not all, medical specialities. However, it seems to be a requirement for success that clinicians have recognised and identified a problem that should be addressed, and that they work together with information technology specialists in integrating the new technology in the established clinical, organisational, and technological systems.

#### Telemedicine is seen as a solution to political and medical issues (category three)

In addition to documenting telemedical applications as solutions to specific local problems (*cf. *categories one and two), successfully implemented telemedical applications often have also been described as advantageous from a health policy or financial perspective. We believe that this indicates that many different people are involved when a telemedical application is implemented, and that the implementation process (and the subsequent description of the process) represents an effort to make the telemedical application valuable and attractive for all those involved.

#### There is collaboration between promoters and users (category four)

In the studies of successful telemedical applications, we have identified three categories of participants: 1) policy entrepreneurs, managers, and bureaucrats, 2) the responsible participants, who are the project leaders, researchers, and designers, and 3) the users, who are the healthcare professionals and patients. The different participants have different perceptions of what telemedicine is, and whether an implementation is to be understood as a success or a failure [13]. In studies of telemedical applications with success, we have seen how the individuals who are responsible for implementing the technology must balance different perceptions during the process of implementation. For example, while the policy entrepreneurs, managers, and bureaucrats understand telemedicine as a general technological solution to structural problems that affect access to health care, the project leaders, designers, and researchers are responsible for translating the optimistic expectancies into project descriptions, system 'thinking', and study protocols. However, in this detailed specification work, the project managers encounter other political expectations, namely expectations that the evidence base for a technology should act to discipline decisions about policy and public spending. The expectations automatically link evaluation and adoption of a technology, and in studies of successful telemedicine, we see the tie-in; all of the studies promise that future evaluations will be conducted, but only after activity in the service increases. It is true that references are made to some effects of the implemented technology, but according to the authors of the studies, it is not possible to make a general statement about the effects precisely because the technology has not been used a great deal in the phase during which it was implemented.

These promises of future evaluation studies reflect an emphasis on integrating the technology into various local practices, and not the accomplishment of randomized controlled trials. Consequently, the studies of successful telemedicine are often descriptions of the process of implementation of a telemedicine application, where it is the implementation itself that is emphasized and not the evaluation activity. We also conclude that telemedical applications in clinical use become more pragmatic and conventional solutions to health care delivery problems than the policy entrepreneurs and bureaucrats initially proposed.

Thus, at the level of technical implementation, the project managers encounter the expectations of the users of the new technology, not the policy makers or the bureaucrats. And, in most of the studies of successfully implemented telemedicine applications, we observed how the local service delivery problem, technical, and organisational structures are mutually shaped during the process of implementation. As mentioned above, we understand that such an approach has been at the expense of producing evidence of the effect of telemedicine. In these studies, the motivation for introducing telemedicine is not evidence of the effects of telemedicine *per se*. Rather, it is a central motivation to increase access to specialist competence. Therefore, it appears that project leaders balance the different perceptions of telemedicine by simply ignoring policy entrepreneurs' and bureaucrats' perceptions of telemedicine during the process of implementation. In the descriptions of the studies' aims, however, it appears that they attempt to accommodate all points of view. It is often unclear whether the intention is to describe the technical specification of the application, the research design and outcome of the application, or the teamwork in the field. The risk of not dedicating the study to one group of participants, or a single perspective, is that the descriptions of the themes become superficial. For readers, whether it is health care professionals who encounter health delivery problems, managers who consider costs related to telemedicine, or project leaders who have become responsible for implementation of telemedical applications, such studies are often of limited use.

#### Issues regarding organisational and technical arrangements are addressed, and the future operation of the telemedical application is considered (categories five and six)

As we mentioned above, technology implementation presumes a style of project management that carefully balances between initiating organizational change and drawing upon technologies as a change agent without attempting to pre-specify and control this process [19]. This style of project management is not explicitly described in the studies of successful implemented telemedical applications, but we assume that it has been present, because of the close collaboration and dialogue between clinicians and those with technical competence. The local clinical context in which the service is to function forms the point of departure for the dialogue, and the telemedical applications need to be adjusted to local clinical needs. Nothing completely new is created, but parts of the activities and general technological possibilities of the local context are mutually formed and reformed in an open and dynamic teamwork relationship. This applies to the problem to be solved, as well as to the methods of solving it. For example, in several of the studies, we see that the telemedicine application has been integrated and adapted to established telemedicine networks [27], or it is a further development of an established service [26]. This type of integration can be seen as a criterion for success, and it requires adequate cooperation between those involved. Cooperation and dialogue promote integration of the technology, but also users' acceptance of, and familiarity, with the technology. Therefore, we conclude that an adopted and integrated application is a feasible application.

In these studies, success relates to the implementation of the technology in a clinical setting, evidence that it functions effectively, and the clinicians' satisfaction with the technology. However, because no evaluation results have been produced about its outcome during the implementation period, a paradoxical situation arises when the implementation is regarded as complete. This means that even though the telemedical application is successful implemented in the sense that it is functional and adapted to its users, it becomes difficult to obtain support for further operation of the application because the positive experience cannot be documented. All the studies therefore end with a comment that more detailed evaluation will be undertaken in the near future. The use of the application is regarded as stable, its activity is expected to increase, making it possible to design evaluation protocols based on the principle of randomized trials. However, the challenge is to obtain funding for further operation because no evaluation results are available. The result is that the activity in the application does not increase and the evaluation cannot be carried out. The outcome of this paradoxical situation is not described in any of the reviewed studies, but we conclude that when such a situation occurs, the clinical involvement may disappear and a potentially successful telemedical application may be discontinued.

In all the studies of successful telemedicine, allowances are made for the fact that the operation of the application must be secured after the implementation activity itself is complete. This is achieved by integrating the new application in established organizational structures as well as creating new ones. As systems for funding the telemedical applications are regarded as vital for continual expansion of use of the application, they should be embedded in this kind of structures. However, only Dolittle [36], Chau and Hu [37] and Khoja *et al. *[38] refers to continued funding of the telemedicine application, but it is unclear whether the funding is legitimated by positive evaluation results. It is also not known how other successful telemedicine examples finance the operation of the service. Unfortunately, funding strategies are not described in any of the reviewed studies.

### Methodological issues

In the present study, we have carried out a qualitative analysis, drawing on principles from GT [20-22,25]. Following the data analysis, we discussed the results in light of STS theory, particularly the concept of 'social negotiation'. While there clearly are different opinions and practices relating to the role of prior research and theory in carrying out GT research [41,42], results obtained by means of GT work may be seen in light of other theories, and that results from GT analyses may be used to discuss other theories.

Few studies describe successfully implemented telemedical applications. These studies also provide little information about the level of activity in the applications when the article was written, and whether they are used at present. In addition, it has been difficult to determine whether some of the studies are descriptions of successful research and development projects that have transitioned into a more or less continuous phase of use, descriptions of successful implementations, so-called implementation projects, or simply descriptions of a telemedical application used in routine health care service. It has been especially challenging to make this distinction if the application has been used for several years, where there has been a strong focus on the implementation issues, where the new service has been incorporated in established telemedicine networks, and whether efforts have been made to encourage use of the application precisely to make it possible to complete the process of evaluations. Examples of such studies are Moses *et al. *[29], Gray *et al. *[27] and LaMonte *et al. *[26].

The criteria that we set up in our methods intended to include only studies of telemedicine in routine use, but because of the difficulties discussed above, we have not been able to define our study unit precisely. The studies mentioned in the discussion above, which may be considered as descriptions of development of telemedical applications and not descriptions of telemedical applications in clinical use, have been included in our qualitative analysis.

Another methodological challenge is that we have only explored characteristics of successful telemedicine applications. That is, we do not know if studies of unsuccessful telemedicine have some of the same qualities as the ones examined here.

Despite the methodological challenges and the scarcity of descriptions of telemedicine used in routine health care services, we conclude that it is possible to make some general statements about the features that characterize successful telemedicine applications. The elements presented in Figure 2 are generalizations, and we have shown how they are implemented in the various studies of successful telemedicine. We conclude that our data suggest the notion that it is the local implementation of these more general issues that represents the real success factor. This is also discussed by Berg [19], Linderoth [40], and May *et al. *[12-14].

### Recommendations

**Figure 3:**
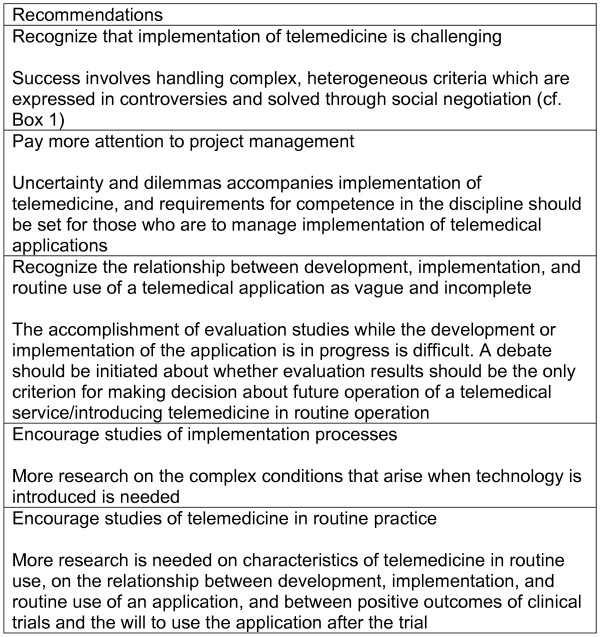
Recommendations.

Successful implementation of telemedicine is no simple matter. The factors presented in Figure 2 may be involved in promoting success, but this success is largely dependent on how these factors is handed when each telemedical application is about to be implemented into clinical practice. The practical implication of such insight is that the implementation and management of telemedical applications must receive more attention. It must be recognized that the mutual adaptation process between technology and problem-solving is difficult to control, as is the outcome. In addition, it would be of great interest to obtain more research-based knowledge about the mutual adaptation process between technology and clinical challenges, the way in which the process has been handled in each case, and the outcome of the approach followed (figure 3).

Based on our work, we conclude that more attention should be given to the differences between telemedical applications used in connection with development and implementation projects as well as between projects and routine use of telemedical applications. We conclude that the distinction between development projects and implementation projects is not seen as a problem, and is therefore not made explicit. By 'development project', we mean a project in which new telemedical application is tested in a clinical context. By 'implementation project', we mean a project in which established telemedical applications is tested in a new clinical context. Development, implementation, and evaluation will necessarily feature in both cases, but the emphasis is different. The transition between development projects, implementation projects, and routine use of telemedical applications merits further research and awareness.

Another issue is the implication of this unclear relationship between development and implementation projects for conducting evaluation studies while the projects are in progress. The lack of evaluation studies may make it difficult to obtain support for further operation of the service. It becomes even more problematic to increase the activity in the service and continue to produce results about its outcome. Therefore, we need to question the relationship between positive evaluations and the diffusion of telemedicine into clinical practice. Should the evaluation results be the only basis for decision-making? No studies that we are aware of have investigated the relationship between positive outcomes of clinical trials and the continued use the service after the trial, nor has the opposite relationship been investigated.

## Conclusion

There have been great expectations for telemedicine, but implementation of such applications has proven to be difficult, and not widely used. It is commonly suggested that the major reason for the low clinical use of telemedicine is the insufficient evidence of its efficacy in terms of both clinical and organizational impact in the health care sector. Without evidence of an effect, professional and political support for telemedicine cannot be sustained. However, lasting recent years, studies on 'organizational issues' have emerged in the field of telemedicine that state that the outcome of clinical trials does not tell the whole story about what is needed to make telemedicine systems and services work.

Our research contributes to this emerging literature. By exploring studies of successful telemedical applications, we have described conditions operating during the process of implementation that are important for its outcome, *i.e.*, what are the characteristics of telemedical services that have been successfully implemented in routine clinical practice. We found that local medical and health-related challenges that the various telemedical applications are intended to solve are clearly described and desired. The teamwork between those who initiate the services and the fields of practice affected is critical. The services and the fields of practice are treated as dynamic quantities that undergo a process of mutual adaptation during the introductory phase of the telemedicine service. The individual burdens that accompany such adaptation processes are recognized. The services are grounded in stable, but flexible organizational and technological structures. The need to secure the future operation of the services is also taken into account.

Our findings support the literature on 'organisational issues' which argues that technologies are not static entities moving from invention through diffusion and into routine use. That is, new technologies alone do not create change. Rather, it is the interplay between technical and social factors that produces particular outcomes. Organizational difficulties lie in the deep interrelationship of technical and social aspects of designing and implementing technologies. Conversely, success involves handling these complex, heterogeneous elements that are expressed in controversies and solved through social negotiation. Practical consequences of our research should be to recognize the uncertainty that accompanies implementation of telemedicine, and more stringent requirements for competence in the discipline should be set for those who are to manage such implementation of the technology. More research on the complex conditions that arise when technology is introduced should be encouraged. Last, but not least, a debate should be initiated in the professional telemedicine community about whether the evaluation result of outcome studies should be the only criterion for introducing telemedicine in routine operation.

## Competing interests

The author(s) declare that they have no competing interests.

## Authors' contributions

AO and KHE have been involved in all stages of the research process. RW has been involved in all stages of the research process except in the initial stage, when the idea was developed and the data were collected. All authors read and approved the final manuscript.

## Supplementary Material

Additional file 1 Appendix. The data provided represent all the articles discussed Click here for file
