# Supplementary material for: Characteristics of successfully implemented telemedical applications
Source: Implement Sci. 2007 Jul 27;2:25. doi: 10.1186/1748-5908-2-25 (PMC1988806; doi:10.1186/1748-5908-2-25)
Supplement: Additional file 1 — Appendix. The data provided represent all the articles discussed [file 1748-5908-2-25-S1.pdf]

Reference	**Local healthcare service delivery problems are clearly stated**	**Telemedicine is seen as a benefit**	**Telemedicine is seen as a solution to political and medical issues**	**There is collaboration between promoters and users**	**Issues regarding organizational and technological arrangements are addressed**	**The future operation of the service is considered**	**Location (L), Participants (P), Type of study (T)**
LaMonte et al. (2003)[26]	Under use of intravenous thrombolytic therapy in areas without local consulting stroke specialist.	Audio-video link; increase the number of patients receiving intravenous thrombolytic therapy.	Improving treatment for acute stroke patients out of university medical centre.	Conversations between actors involved; planning meetings accomplished; training sessions and ICT support arranged; evaluation performed.	Telemedical application emanated from a traditional telephone consultation service; routines for training sessions established; guidelines for use prepared.	Further studies on outcome, quality, and effect. Lack of funding and reimbursement mechanisms will limit the development in this field.	L: Baltimore, USA. University medical centre and community hospital.<br>P: 50 telemedicine/telephone consultations.<br>T: Description of service.
Gray et al. (1998) [27]	Need for increased information and support to families of high risk newborns during hospitalization and following discharge.	Baby-CareLink; gives access to personal information from any computer with a standard web browser.	Improve service provided by the hospital to families of high risk newborns.	Conversations between actors involved; training sessions and ICT support arranged; evaluation performed.	Evaluation study in progress.	The service has been integrated into existing organisational structures.	L: Boston, USA.<br>P: High risk neonates <1500 grams.<br>T: Description of service, no outcomes at this stage.
Ono and Lindsey (2004)[28]	Deficient access to health care for children with paediatric	Tele-peadiatrics network: real time specialist	Enhance access to specialist health care in rural areas.	Motivated and determined actors involved;	Harmonizing with an initiative to develop a	To overcome health care personnel's "techno-fear"	L: Honolulu, Hawaii and other islands in the Pacific.

	orthopaedic conditions or need for plastic surgery in the Islands in the Pacific Sea.	consultations.		medical director and telemedicine coordinator appointed, networking towards the remote sites and funding providers.	comprehensive state wide telemedicine network in Hawaii		P: >240 consultations.<br>T: Description of service.
Moses et al. (1997) [29]	High cost involved in transporting specialists in order to perform routine endoscopies in rural areas.	Tele-endoscopy; support rural general surgeons and gastro-enterologists in performing endoscopies locally.	Enhance accessibility to quality health care in rural areas.	Evaluation study performed.	Telemedical application as part of a state wide telenetwork; continuance of the evaluation.	Remember the importance of essential and prospective evaluation of new applications.	L: Vermont, USA.<br>3 pilot studies; a) 30 patients – image quality/diagnostic concordance study.<br>b) Chart review, study of cost-effectiveness.<br>c) Interviews with 4 potential users (doctors).
Gulube and Wynchank (2001) [30]	Lack of health services in rural areas of South Africa.	Tele-radiology, tele-ultrasound program; improve primary health care	Mitigate perceived differences in quality of health care caused by former political regime.	Political, commercial, and scientific actors cooperate designing, coordinating, and	Establishing a national telemedicine system; continuation of evaluation programs; guidelines for	Challenges regarding technical and organisational issues have been considered	L: South Africa.<br>28 pilot sites.<br>P: 264 teleradiology sessions, 9 antenatal ultrasound consultations.

		services and health education.		implementing a telemedicine program; evaluation performed	use prepared		T: Description of service. Outcomes: reduction in patient transfers.
Chan et al. (2001) [31]	Need to Improve prenatal care in rural areas of Queensland.	Tele-ultrasound; improve access to prenatal care; reduce travelling time; improve access to medical education.	Reduce health care gap between urban and rural areas; increase access to specialist treatment.	Collaboration between promoters and users; evaluation performed.	The telemedical application has become an integrated part of ordinary clinical practice.	Limitations to further diffusion of the application has been considered.	L: Queensland, Australia. P: 71 patients + clinicians, It/technical staff, managers T: Quantitative. Technical and clinical evaluation (satisfaction and diagnostic impact).
Lawton et al. (2004) [32]	Increase in waiting time for dermatology outpatient appointments in Nottingham.	Tele-dermatology; provide diagnosis efficiently, improve treatment and management of derma-tological conditions in primary care.	Deliver modern patient-centred services in enhanced and accessible surroundings; working across traditional boundaries.	Involvement and commitment between actors involved; evaluation performed.	Telemedical application viewed as an integral part of the community medical service; establishment of a teledermatology team; treatment protocols and referral pathways into	n/a	L: Nottingham, UK. A hospital dermatology department and 4 primary care trusts. P: GP, nurses, patients. T: Description of service.

					secondary care established.		
DiLieto et al. (2002) [33]	Need for increased specialised medical expertise to pregnant women in rural areas of Italy.	Tele-cardio-tocography: increase quality of care to pregnant and newborn; reduce pregnant women's need for stressful travelling and/or hospital admission.	Give near term pregnant women in rural areas the same access to high quality CTG examinations as women in urban areas.	Collaboration between promoters and users; local user resistance discussed; evaluation performed.	Guidelines for use established.	Obstacles between different health care providers have been considered.	L: Naples, Italy, and 5 centres 15-30 km away.<br>P: 162 patients, medical/technical staff.<br>T: Description of service.<br>Outcomes: no of C-sections, users' satisfaction.
Lin et al. (2001)[34]	Poorly distributed medical resources in rural areas of Taiwan.	Telemedicine system (primarily tele-radiology); reduce frequency of referrals; provide CME opportunities.	Improve medical services in rural areas.	Evaluation accomplished; system adjusted to user requirements.	Continuing development of technology.	Further development of web-based telemedicine	L: Taiwan.<br>3 hospitals + local health care centre.<br>P: 1107 consultations.<br>T: Description of service.<br>Outcomes: change of diagnosis, transferrals, satisfaction.
Urness (1999) [35]	Deficient access to health care for patients with	Tele-psychiatric service;	Removal of geographic barriers to	Cooperation and commitment	Dedicated administrative unit defined;	n/a	L: Alberta, Canada. One major and 5 rural

	psychiatric disease in rural areas of Alberta.	enhance access to specialist care; reduce travel time for patients and staff; reduce waiting time.	specialty mental health consultation.	between actors involved regarding administration of the program and funding; training session and ICT support arranged; evaluation performed; gradual expansion of the program.	integration with ordinary service; equipment made available for other purposes; evaluation planed.		hospitals linked.<br>P: 109 consultations.<br>T: Description of service.<br>Outcomes: acceptance/satisfaction. Per cent of consultations that are follow-ups.
Doolittle (2001) [36]	Need for increased access to specialist health care in remote areas of Kansas.	Educational tool; provide access to specialist care and consultations (oncology, paediatrics); provide short response time for urgent cases; reduce travel time; increase recruitment	Increase access to specialist health care at geographically remote areas.	Telemedicine coordinator appointed; commitment and participation between actors involved; gradual expansion of program.	Goals and guidelines for use established; routines for financial support established.	The successful parts of the service have been integrated into existing organisational structures.	L: Kansas, USA. University medical centre and >40 sites.<br>P: Several programmes; 170 tele-oncology patients, >1000 school-based tele-paediatric consultations, etc.<br>T: Description of service. Reports of rates of satisfaction from

Doolittle (2001) [36]	Need for increased access to specialist health care in remote areas of Kansas.	Educational tool; provide access to specialist care and consultations (oncology, paediatrics); provide short response time for urgent cases; reduce travel time; increase recruitment and retention of local GPs.	Increase access to specialist health care at geographically remote areas.	Telemedicine coordinator appointed; commitment and participation between actors involved; gradual expansion of program.	Goals and guidelines for use established; routines for financial support established.	The successful parts of the service have been integrated into existing organisational structures.	L: Kansas, USA. University medical centre and >40 sites.<br>P: Several programmes; 170 tele-oncology patients, >1000 school-based tele-paediatric consultations, etc.<br>T: Description of service. Reports of rates of satisfaction from medical/nursing staff, patients/parents.
Chau and Hu (2004) [37]	Need to increase quality of diagnosis and treatment to patients with neurosurgical conditions in Hong Kong.	Telemedical application; neurosurgeons at an acute tertiary hospital provide remote consultations to physicians. at a regional general	Achievement of vertical integration of patient care and management services	A motivated and determined clinical administrator and motivated surgeons.	The telemedical application has become part of a Hong Kong-based telemedicine program; guidelines for use are established.	The service has been integrated into an accounting system.	L: Hong-Kong<br>P: 2-3 transmissions per day, including surgeons and technical staff.<br>T: Qualitative, case-study, interviews with those involved.

Kavanagh and Hawker (2001) [39]	Need to improve the mental health services in rural South Australia.	Tele-psychiatry service; videoconference consultations; provide effective and reliable psychiatric treatment to patients in distant locations.	Enhance access to quality health care in rural areas.	Continuous expansion and improvement of the tele-psychiatry service.	Guidelines for use of the tele-psychiatry service established; management support established.	The service has been integrated into existing organisational structures.	L: Adelaide, Australia, and 48 peripheral sites.<br>P: Ca 100 sessions/month.<br>T: Description of service. Outcome: continuation of service.
Johnson (2004) [43]	A rural office of Vocational Rehabilitation offers limited services when working with clients who are deaf or hard of hearing.	Audio-video network; Improve overall employment outcomes for clients who are deaf and hard of hearing.	Mitigate perceived regional differences in quality of services caused by distances.	Building alliances and partnership between actors involved; planning meetings accomplished; evaluation performed.	Continuance of the evaluation.	A telemedicine state program is established.	L: Utah, USA. 4 vocational rehabilitation centres + 10 postsecondary locations.<br>P: Staff, sign-language interpreters.<br>T: Description of service, outcome: users' satisfaction.
Schneider (2004)[44]	High rate of re-hospitalization in relation to congestive heart failure; loss of	Home-monitoring; transferring vital signs to a home care	Improve patient care; reduce the number of re-hospitalizations; decrease	Cooperation between actors involved when	There is a dedicated pool of staff operating the system.	n/a	L: Pennsylvania, USA.<br>P: unknown number of patients.

	revenue for home care agencies and hospitals.	centre.	financial losses.	choosing application; motivation of staff; training session for staff and patients arranged.			T: Description of service. Outcomes: reduction in number of home nursing visits and rehospitalisation rates.
